# Capping protein integrates multiple MAMP signalling pathways to modulate actin dynamics during plant innate immunity

**DOI:** 10.1038/ncomms8206

**Published:** 2015-05-28

**Authors:** Jiejie Li, Jessica L. Henty-Ridilla, Benjamin H. Staiger, Brad Day, Christopher J. Staiger

**Affiliations:** 1 Department of Biological Sciences, Purdue University, 335 Hansen Life Sciences Building, West Lafayette, Indiana 47907-2064, USA; 2 Department of Plant, Soil and Microbial Sciences, Michigan State University, East Lansing, Michigan 48824-6254, USA; 3 The Bindley Bioscience Center, Discovery Park, Purdue University, 1203 West State Street, West Lafayette, Indiana 47907, USA

## Abstract

Plants and animals perceive diverse microbe-associated molecular patterns (MAMPs) via pattern recognition receptors and activate innate immune signalling. The actin cytoskeleton has been suggested as a target for innate immune signalling and a key transducer of cellular responses. However, the molecular mechanisms underlying actin remodelling and the precise functions of these rearrangements during innate immunity remain largely unknown. Here we demonstrate rapid actin remodelling in response to several distinct MAMP signalling pathways in plant epidermal cells. The regulation of actin dynamics is a convergence point for basal defence machinery, such as cell wall fortification and transcriptional reprogramming. Our quantitative analyses of actin dynamics and genetic studies reveal that MAMP-stimulated actin remodelling is due to the inhibition of capping protein (CP) by the signalling lipid, phosphatidic acid. In addition, CP promotes resistance against bacterial and fungal phytopathogens. These findings demonstrate that CP is a central target for the plant innate immune response.

## Abstract (web summary)

Cytoskeletal remodelling is an important component of the innate immune response in plants. Here, Li *et al*. demonstrate that pathogen-triggered actin remodelling is due to the inhibition of capping protein (CP), and show that CP is required for resistance against plant pathogens.

## Graphical abstract

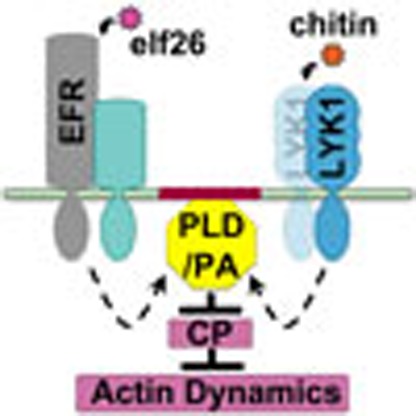

Both animals and plants use innate immune signalling mechanisms to defend against microbial infection. Pattern recognition receptors (PRRs) sense pathogen- or microbe-associated molecular patterns (MAMPs) and activate the first layer of innate immune responses^1^,^2^. In mammalian cells, Toll-like receptors are the major PRRs to perceive MAMPs^3^. Activation of Toll-like receptors triggers dynamic cellular changes to resist pathogen attack, which often depend on actin remodelling to rearrange receptors and cell-signalling intermediates, and to mediate membrane movements in processes such as endocytosis, plasma membrane ruffling and phagocytosis^3^.

In contrast to mammals, plants rely exclusively on innate immunity of each cell to recognize and respond to signals upon microbial challenge. This is because they lack adaptive immunity and mobile immune cells specialized in microbial recognition^2^. Moreover, plants have evolved a large repertoire of PRRs for recognition of diverse MAMPs from both beneficial and pathogenic microbes^4^. Several well-characterized MAMP/PRR pairs have been described in *Arabidopsis.* Bacterial flagellin, or the peptide mimic flg22, is recognized by the leucine-rich repeat receptor kinase, FLAGELLIN-SENSING2 (FLS2; ref. 5). The conserved amino terminus of bacteria elongation factor EF-Tu, represented by peptides elf18 or elf26, is recognized by another leucine-rich repeat receptor kinase, EF-TU RECEPTOR (EFR; ref. 6). The β-1,4-linked GlcNAc chitooligosaccharide, chitin, from fungal cell walls interacts with several lysin motif (LysM) receptor-like kinases, including CERK1/LYK1 and LYK5 (refs 7, 8, 9, 10). The recognition of different MAMPs by specific PRRs induces common signalling events involving activation of mitogen-associated and calcium-dependent protein kinases (MAPK and CDPK), bursts of cytosolic calcium and reactive oxygen species (ROS), as well as extensive transcriptional reprogramming^4^,^11^,^12^. Cellular immune responses, such as rearrangement of cytoplasmic organelles and cell wall reinforcement by callose deposition, are also activated to abrogate the pathogen infection^4^,^12^,^13^,^14^.

Remodelling of the actin cytoskeleton during the innate immune response has been observed in a broad range of plant–microbe interactions. For example, actin bundles form at the attack or entry site of fungi and oomycetes^15^. In symbiotic interactions, invading bacteria or purified Nod factors lead to the rearrangement of actin arrays in root hairs, perhaps by increasing the availability of barbed ends of actin filaments^16^,^17^. Moreover, a recent study from *Arabidopsis* showed that both pathogenic and nonpathogenic bacteria elicit a transient increase in the density of actin filament arrays in epidermal pavement cells^18^. In addition, treatment with MAMPs was sufficient to stimulate an increase in filament abundance^18^. Many defence responses, such as cytoplasmic rearrangements and targeted delivery of defence compounds to the infection site, are dependent on actin remodelling^15^,^19^. For example, the targeting of *Arabidopsis* resistance protein RPW8.2 and the ABC transporter protein PEN3 to the host–cell plasma membrane requires a functional actin cytoskeleton^19^,^20^. Furthermore, cell wall fortification by callose deposition is significantly impaired when the host actin cytoskeleton is disrupted^19^,^21^,^22^. Actin remodelling and associated cellular processes are thus important facets of the response to microbes; when the actin cytoskeleton is perturbed, plants are more susceptible to both pathogenic and nonpathogenic microbes^15^,^18^,^23^,^24^. Collectively, these findings suggest that the actin cytoskeleton is not only a key target of innate immune signalling in perception of both beneficial and pathogenic microbes but also integrates the signalling to further elicit broad cytological defence responses. However, the molecular mechanisms that underpin actin remodelling and the precise functions of these rearrangements in host defence responses remain to be fully elucidated.

Recently, the changes in actin organization and dynamics during innate immune signalling have been examined at high spatial–temporal resolution in living *Arabidopsis* cells^22^. In response to signalling stimulated by elf26, actin filament abundance in hypocotyl epidermal cells increases within minutes^22^. Further analyses of single-filament dynamics suggest that the increased filament abundance results from decreased filament disassembly via the inhibition of a severing protein—ACTIN DEPOLYMERIZING FACTOR4 (ADF4; ref. 22). In addition, treatment with elf26 leads to increased availability of filament ends for assembly, indicated by markedly enhanced filament–filament annealing^22^. However, in an *adf4* mutant, the frequency of filament–filament annealing still increases to a level that is comparable with wild type (WT) following elf26 treatment, suggesting that additional regulators are required to control the availability of actin filament ends during innate immune signalling^22^. Moreover, the ADF4 pathway may be limited in scope, since *adf4* mutants have a WT response for actin remodelling when challenged with the fungal MAMP, chitin^22^.

Capping protein (CP) is a ubiquitous regulator of actin filaments^25^,^26^. It binds the barbed end of actin filaments to prevent actin polymerization and filament–filament annealing *in vitro*^25^,^27^. Genetic evidence from various organisms demonstrates that the availability of barbed ends for actin assembly is inversely correlated with cellular CP levels^28^,^29^,^30^. In addition, CP has been implicated in the crosstalk of signalling events from membrane to actin cytoskeleton via phospholipid binding^31^. Plant CP is unique in the ability to bind the stress signalling phospholipid, phosphatidic acid (PA), which inhibits capping activity and uncaps filament ends *in vitro* and *in vivo*^26^,^27^,^29^,^31^. A role for CP in the innate immune response of plant or animal cells has not been established, however. Here, we provide the first genetic and pharmacological evidence that negative regulation of CP by PA is essential for actin remodelling during plant innate immune signalling.

## Results

### Actin filament abundance increases in response to MAMPs

Epidermal cells from hypocotyls of dark-grown *Arabidopsis* seedlings provide excellent material for imaging cytoskeletal dynamics during the response to environmental cues^32^,^33^. Previously, we found that rearrangement of cortical actin arrays in hypocotyl cells occurs within minutes following treatment with the bacterial MAMP elf26 (ref. 22). Another MAMP, flg22, fails to induce actin remodelling because the receptor *FLS2* is not expressed in dark-grown seedlings of *Arabidopsis*^22^. To examine whether actin arrays respond to additional MAMPs, we tested chitin. Dark-grown seedlings expressing the actin reporter GFP-fABD2 (refs 32, 34) were treated with 1 μM MAMP for 5 min, and static images of the cortical actin cytoskeleton were collected by variable angle epifluorescence microscopy (VAEM).

**Figure 1:**
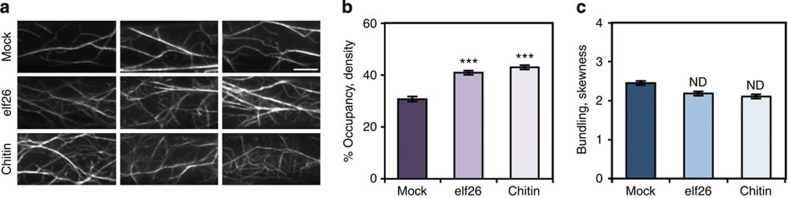
Actin filament abundance increases in response to MAMPs. (**a**) Representative VAEM images of hypocotyl epidermal cells expressing GFP-fABD2 from WT seedlings grown in continuous darkness for 5 days. Seedlings were treated for 5 min with mock, 1 μM elf26 or 1 μM chitin before imaging. Scale bar, 10 μm. (**b**) Average filament density, or percentage of occupancy, analysis was performed on images collected from epidermal cells in the basal third of hypocotyls. When compared with mock-treated cells, the actin filament density was significantly increased after 1 μM elf26 and 1 μM chitin treatments. (**c**) The extent of filament bundling, or skewness, was measured on the images used for **b**. No significant differences were observed among treatments. Values given are mean±s.e.m. (*n*⩾200 cells from 10 hypocotyls per treatment; ***P<0.001; ND, no significant difference; Student's *t*-test).

Following chitin treatment, the cortical actin array in epidermal cells was noticeably more dense than in mock-treated cells (Fig. 1a). To quantify cytoskeletal remodelling, the architecture of cortical actin arrays was measured with a set of metrics described previously^29^,^35^,^36^,^37^. Skewness and density are metrics used to estimate the extent of actin filament bundling and the percentage of occupancy of actin filaments, respectively. As shown in Fig. 1b, actin filament abundance in the cortical array was significantly increased after 5-min treatment with 1 μM elf26 or chitin, which is consistent with previous results^22^. Moreover, the cytoskeletal response to chitin treatment was both dose- and time dependent (Supplementary Fig. 1a,c). No significant changes in the extent of filament bundling were observed when cells were treated with concentrations of chitin ≤1 μM or for periods of time <15 min (Fig. 1c and Supplementary Fig. 1b,d). However, treatment with 10 μM chitin for 5 min, or with 1 μM chitin for ⩾15 min, led to reduced filament bundling (Supplementary Fig. 1b,d). Collectively, these data demonstrate that the cortical actin array in hypocotyl epidermal cells responds within minutes to several diverse MAMPs leading to a significant increase in actin filament abundance.

### Actin remodelling requires perception of MAMPs by cognate PRRs

During the plant defence response, initial signalling steps are activated upon the perception of MAMPs by PRR complexes located on the cell surface^4^. In *Arabidopsis*, elf26 is perceived by EFR^6^. For chitin recognition, two LysM-containing receptor proteins have been characterized^7^,^8^,^9^,^10^. LYK1 (also named CERK1) was previously described as a PRR for sensing chitin^7^,^8^, whereas LYK4 is also involved in, but not essential for, chitin recognition^9^. Recently, LYK5 was shown to be a major receptor for chitin in *Arabidopsis*^10^. Here we focus our attention on *lyk1* and *lyk4* single and double mutants. Both *LYK1* and *LYK4* were found to be expressed in dark-grown seedlings, with transcript levels for *LYK1* markedly more abundant than *LYK4* (Supplementary Fig. 2).

**Figure 2:**
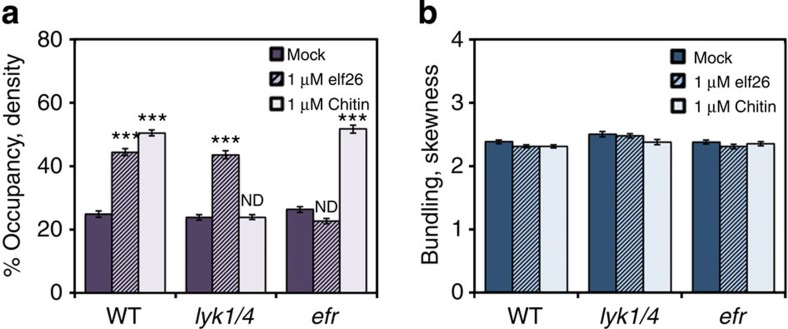
The increase in actin filament abundance following chitin elicitation requires the LYK receptor. (**a**) Analysis of filament density was performed on *Arabidopsis* WT or mutant hypocotyls treated with mock, 1 μM elf26 or 1 μM chitin for 5 min. WT epidermal cells after elf26 or chitin treatments had a significant increase in filament abundance. Treatment with elf26 increased the filament abundance in *lyk1 lyk4* double mutant cells, however, chitin treatment did not. The actin arrays in *efr* mutant cells were significantly more abundant following chitin treatments, but were unresponsive to elf26 treatments. (**b**) The extent of filament bundling was measured on the images used for **a**. No significant change in filament bundling following treatment with MAMPs was observed compared with respective mock controls. Values given are mean±s.e.m. (*n*⩾200 cells from 10 hypocotyls for each treatment and genotype; ***P<0.001 compared with respective mock control; ND, no significant difference; Student's *t*-test).

To test whether actin cytoskeleton remodelling in response to elf26 and chitin requires cognate PRRs, we quantified the actin architecture in homozygous *lyk* knockout mutants, as well as in an *efr* mutant. As expected, the increase in actin filament abundance following chitin treatment was impaired in *lyk* mutants (Fig. 2a and Supplementary Fig. 3). In cells of the *lyk1* single mutant treated with chitin, there was an almost complete failure of the actin density increase, whereas the *lyk4* single-knockout mutation reduced, but did not eliminate, the cytoskeletal response to chitin (Supplementary Fig. 3). This suggests that LYK1 plays a major role in chitin-elicited actin remodelling in dark-grown hypocotyls. Moreover, in a *lyk1 lyk4* double mutant, the density of actin arrays was completely unresponsive to chitin treatment (Fig. 2a). By contrast, elf26 treatment led to an increase in actin filament abundance in all *lyk* single and double mutants to levels that were comparable with WT (Fig. 2a and Supplementary Fig. 3a). As expected, the density of actin arrays in the *efr* mutant did not increase in response to elf26 treatment, but was significantly increased following chitin treatment (Fig. 2a)^22^. No significant changes to the extent of actin filament bundling were observed following either elf26 or chitin treatment for any of the PRR mutants (Fig. 2b and Supplementary Fig. 3b)^22^. Collectively, these results suggest that actin remodelling in response to diverse MAMPs requires the early signalling events triggered by cognate PRR complexes.

### Dynamicity of actin array is enhanced transiently by MAMPs

**Figure 3:**
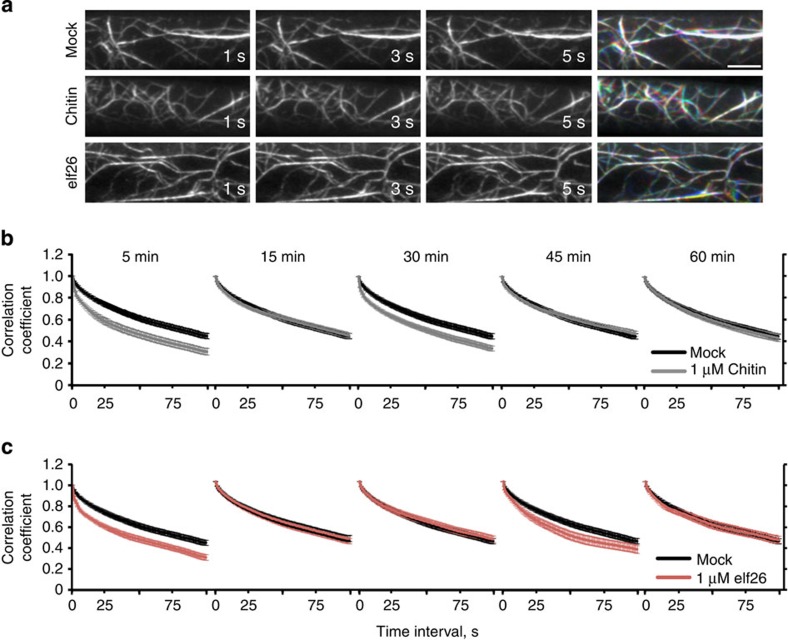
Dynamicity of the cortical actin array is enhanced by MAMPs. (**a**) Time-lapse VAEM images from hypocotyl epidermal cells following mock, 1 μM elf26 and 1 μM chitin treatments for 5 min are shown. Three images separated by 2 s were coloured red, green and blue, respectively, and the three images merged in the right column. A white colour indicates actin structures that remain relatively stationary during this time period. Scale bar, 10 μm. (**b**,**c**) A correlation coefficient analysis was performed on time-lapse VAEM series from WT epidermal cells treated with mock, 1 μM chitin (**b**) or 1 μM elf26 (**c**) for 5, 15, 30, 45 and 60 min. The extent of actin rearrangements during a given time period, or overall dynamicity of the actin array, was determined by decay in correlation as the temporal interval increased. Lower correlation values correspond with higher dynamicity of the actin array. When compared with mock control, the actin array dynamics were significantly enhanced in cells treated with chitin for 5 or 30 min (**b**); *P*<0.0001; analysis of variance (ANOVA). However, the dynamicity remained similar to mock control following treatments with chitin for 15, 45 or 60 min (**b**); *P*>0.05; ANOVA. Cells treated with elf26 for 5 or 45 min showed significantly enhanced dynamicity compared to mock controls (**c**); *P*<0.0001; ANOVA. No significant differences in actin array dynamicity between mock controls and elf26 treatments were observed at the 15, 30 and 60 min time points (**c**); *P*>0.05; ANOVA. Analyses were performed on >40 time-lapse series taken from 10 hypocotyls for each treatment per time point. Error bars represent s.e.m.

The cortical actin array in WT hypocotyl epidermal cells undergoes constant remodelling^32^,^33^. By merging images from three consecutive time points in a colour overlay, these rapid changes in actin organization can be assessed qualitatively (Fig. 3a). We observed more robust actin dynamics following 5-min treatment with elf26 or chitin, when compared with mock control (Fig. 3a). To better understand the kinetics of actin remodelling, we quantified the overall dynamicity of cortical actin arrays during a 1-h time course of MAMP treatment. Correlation coefficient analysis provides a global view of changes in actin organization over time by calculating the correlation of the intensity of GFP-fABD2 signal at all pixel locations between all temporal pairs of images^38^. The correlation coefficient values from actin arrays with enhanced rearrangement will decay more quickly than a control population. At each time point after MAMP treatment (for example, 5, 15, 30, 45 and 60 min), time-lapse series of 100 frames at 1 s intervals were collected from >40 epidermal cells. In cells treated with elf26 and chitin, the overall dynamicity of actin arrays was significantly increased at two distinct time points (elf26: 5 and 45 min; chitin: 5 and 30 min; Fig. 3b,c). At all other time points tested, MAMP-treated cells showed no difference compared with mock-treated controls (Fig. 3b,c). These data demonstrate that, in addition to actin organization, overall actin array dynamics were enhanced in response to diverse MAMPs. Moreover, the transient nature of the increase in dynamicity of actin arrays indicates that there might be distinct MAMP-elicited signalling events impinging on actin cytoskeleton remodelling at different time points. In the remainder of this study, we focus on the initial actin response that occurs at 5 min after MAMP treatment.

### Individual actin filament dynamics are altered by MAMPs

**Figure 4:**
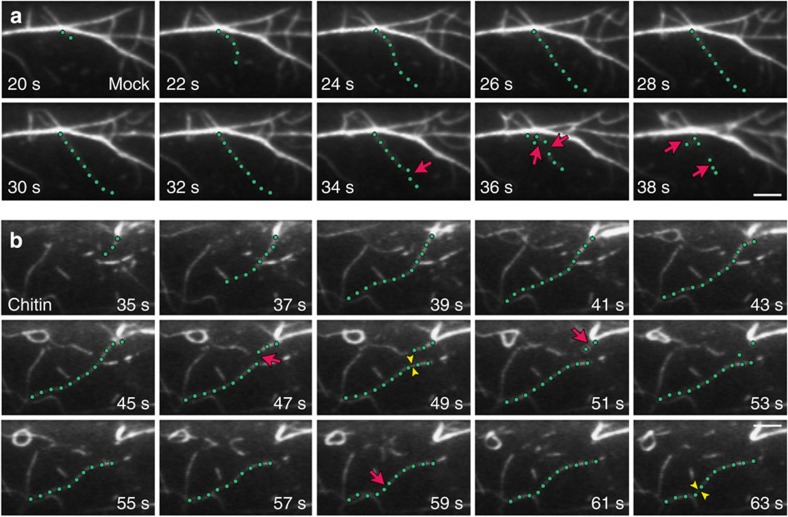
The dynamic behaviour of individual actin filaments is altered in response to chitin. (**a**) A representative actin filament (green dots) from a mock-treated epidermal cell elongated rapidly and was subsequently disassembled by numerous severing events (red arrows). Scale bar, 5 μm. (**b**) A representative actin filament (green dots) from an epidermal cell treated with 1 μM chitin for 5 min elongated rapidly and persists throughout the total elapsed time. Very few severing events occurred during the time course (red arrows). Filament–filament annealing (yellow arrowheads) was also prevalent, with two annealing events observed for this single filament. Scale bar, 5 μm.

An increase in actin filament abundance could occur through multiple distinct and/or overlapping mechanisms^33^. To investigate the underlying molecular mechanisms, we examined the behaviour of individual actin filaments following treatment with MAMPs. In mock-treated epidermal cells, new growing filaments originate from the following three locations: *de novo* in the cytoplasm, the ends of pre-existing fragments and the side of existing filaments or bundles. The proportion of these nucleation events is roughly equal (34%/22%/44%; *de novo*/ends/side; Supplementary Table 1 and ref. 33). After initiation, filament barbed ends elongate at an average rate of ∼1.7 μm s^−1^, and growing filaments reach a mean maximum length of ∼12 μm (Fig. 4a and Supplementary Table 1). These growing filaments usually persist in the cytoplasm for ∼20 s before they are disassembled by severing activity (Fig. 4a and Supplementary Table 1). Filament severing occurs at a frequency of 0.015 breaks per μm s^−1^ in mock-treated cells (Supplementary Table 1 and ref. 33). The availability of barbed ends is also precisely controlled. Only ∼3% of the newly created barbed ends in untreated cells can anneal or regrow (Supplementary Table 1 and ref. 29).

Within 5 min, changes in the behaviour of individual actin filaments were observed following elf26 (ref. 22) or chitin treatment (Fig. 4b and Supplementary Table 1). Only a subset of the dynamic parameters was altered after MAMP treatment; specifically, there was a significantly increased filament length (∼18 μm) and lifetime (∼25 s), as well as reduced severing frequency (Supplementary Table 1). Moreover, the frequency of filament–filament annealing was increased up to fourfold by MAMP treatment (Supplementary Table 1). The proportion of filament origins was also altered in cells treated with MAMPs, with side nucleation occurring preferably after elf26 treatment, and increased filament origin from ends following chitin treatment (Supplementary Table 1). Treatment with elf26 or chitin, however, did not affect the filament elongation rate or the regrowth frequency of newly created ends (Supplementary Table 1). This quantitative analysis of dynamic parameters for single filaments demonstrates that a MAMP-induced increase in actin filament abundance results from reduced filament disassembly, as well as enhanced availability of dynamic ends.

### Actin organization in *cpb-1* mutant is insensitive to MAMPs

The changes in actin organization and dynamics after MAMP treatment appear to phenocopy a knockdown mutant for CP, a filament barbed-end binding protein^29^,^30^. Specifically, reduction of CP in *Arabidopsis* hypocotyl epidermal cells leads to more dynamic filament ends and this significantly enhances filament–filament annealing. In addition, filament dynamics are changed in *cp* mutants, with a significant increase in maximum filament length and lifetime, as well as an apparent decrease in severing frequency. Consequently, the altered dynamic properties of actin filaments result in a more dense cortical array in epidermal cells^29^,^30^. Given that the MAMP-elicited increase in actin filament abundance appears to be a consequence of the enhanced availability of dynamic ends as well as reduced filament disassembly, we hypothesize that CP is negatively regulated or inhibited during innate immune signalling. Thus, we expect that enforced reduction of cellular CP levels should influence actin organization in non-elicited plants as MAMP application does.

**Figure 5:**
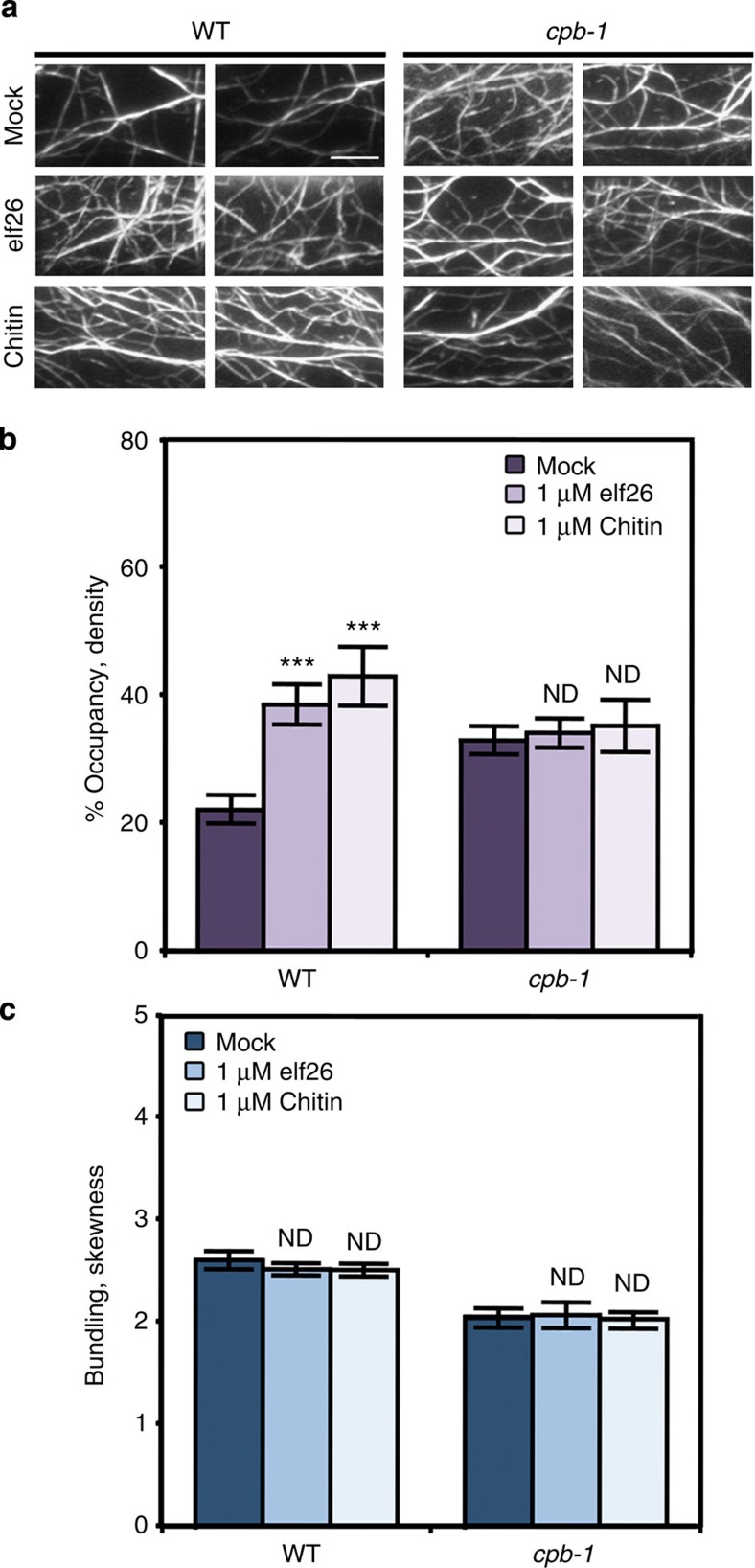
Actin remodelling in *cpb-1* mutant cells is insensitive to MAMPs. (**a**) The cortical actin array in mock-treated *cpb-1* mutant cells was more dense compared with WT cells, as shown previously^29^,^30^. An increase in the density of the cortical actin array was observed in WT cells following treatment with MAMPs. In contrast, actin organization in *cpb-1* mutant cells remained similar between mock and MAMP treatments. Scale bar, 10 μm. (**b**,**c**) Actin architecture analysis was performed on epidermal cells of WT and *cpb-1* mutant treated with mock and MAMPs for 5 min. Unlike WT cells, where actin filament abundance was significantly enhanced following MAMP treatment, no significant change in actin filament abundance was detected in *cpb-1* mutant cells treated with elf26 or chitin compared with mock control (**b**). The extent of filament bundling was not altered in WT and *cpb-1* mutant cells after MAMP treatments compared with their respective mock controls (**c**). Values given are mean±s.e.m. (*n*⩾200 cells from 10 hypocotyls for each treatment and genotype; ***P<0.001; ND, no significant difference compared with mock control of the same genotype. Student's *t*-test).

To test this prediction, we applied elf26 or chitin to *cpb-1* mutant seedlings and compared actin architecture quantitatively. As shown in Fig. 5, actin arrays in *cpb-1* mutant cells were more dense and less bundled compared with mock-treated WT cells, similar to our previous findings^29^,^30^. Following elf26 or chitin treatment, a significant increase in the density of actin filament arrays was observed in WT cells (Fig. 5a,b). The organization of actin arrays in the *cpb-1* mutant, however, did not show any significant differences when mock was compared to treatment with either MAMP (Fig. 5). Another knockdown mutant allele, *cpb-3*, also failed to respond when treated with elf26 or chitin, confirming that the phenotype is due to a reduction in CP levels (Supplementary Fig. 4).

**Figure 6:**
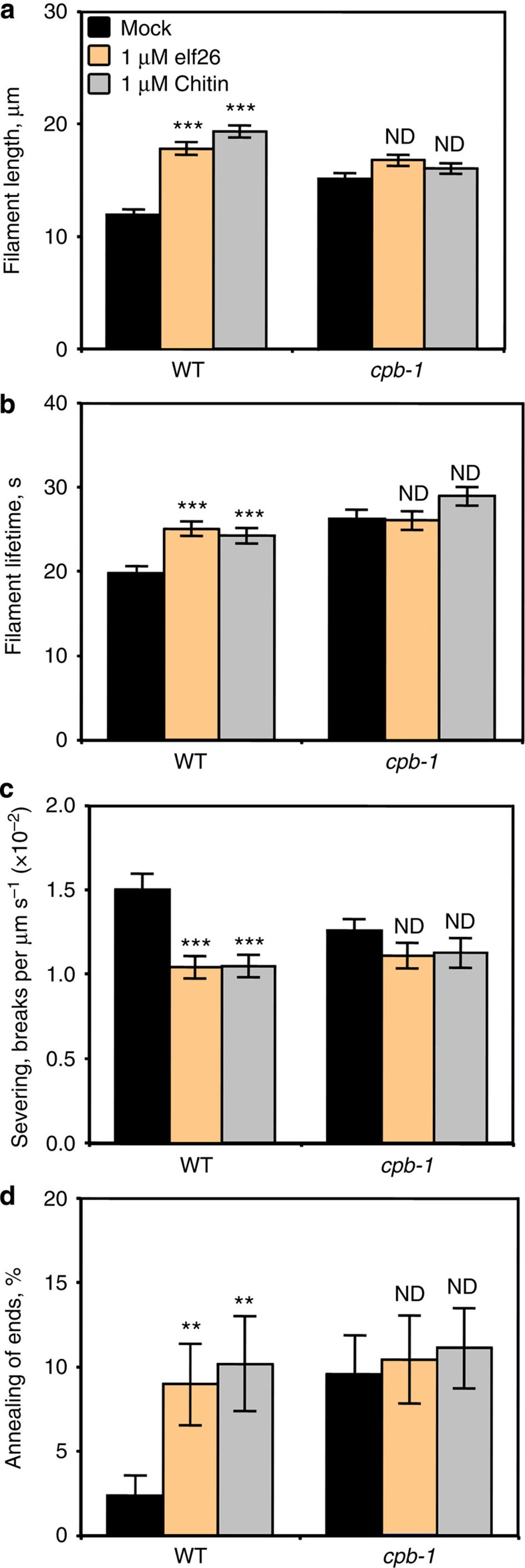
Individual actin filament dynamics in *cpb-1* mutants are insensitive to MAMPs. Actin filaments in WT cells treated with MAMPs showed significantly increased maximum length and lifetime (**a**,**b**), as well as a reduction in severing frequency (**c**). The frequency of filament–filament annealing was also enhanced in WT cells after MAMP treatment (**d**). However, MAMP treatments did not impact any of these parameters in *cpb-1* mutant cells. See also Supplementary Table 1, for full analyses of single-filament parameters. Values given are mean±s.e.m., with *n*>50 filaments from at least 10 hypocotyls per treatment (**P<0.01, ***P<0.001; ND, no significant difference compared with mock control from the same genotype; Student's *t*-test).

To further confirm that inhibition of CP contributes to an increase in filament abundance during innate immune signalling, we examined the behaviour of single actin filaments in *cpb-1* mutant epidermal cells following elf26 and chitin treatment. As shown in Fig. 6 and Supplementary Table 1, the changes in filament dynamic behaviour induced by MAMP treatment were abrogated in the *cpb-1* mutant. In particular, the frequency of filament–filament annealing remained similar between mock and MAMP treatment in the *cpb-1* mutant (Fig. 6d). Moreover, the maximum filament length and lifetime did not increase, and the severing frequency did not decrease in *cpb-1* cells treated with MAMPs (Fig. 6a–c). Collectively, these data provide genetic evidence that the negative regulation of CP is sufficient for MAMP-induced actin remodelling in *Arabidopsis* epidermal cells. Furthermore, due to a reduction of CP levels, actin organization and dynamics fail to respond to either elf26 or chitin treatment, suggesting that CP integrates signals from multiple PRR complexes, and transduces these signalling events into changes in actin cytoskeleton behaviour.

### PA production is required for MAMP-induced actin remodelling

Biochemically, the signalling lipid PA has been shown to bind and inhibit the activity of recombinant CP^27^. Our previous studies show that addition of exogenous PA at concentrations of 10–100 μM leads to more abundant cortical actin arrays in a dose-dependent manner, whereas the control treatment with phosphatidlyserine at the highest concentration tested (100 μM) has no significant effect on actin organization^29^. The increased actin filament abundance after PA treatment recapitulates the actin remodelling that occurs following MAMP treatments. In addition, it can be inferred that the PA-induced increase in filament abundance results from inhibition of CP, since the response to exogenous PA treatment is ameliorated in the *cpb-1* mutant (Supplementary Fig. 5 and refs 29, 31). These data suggest that the rapid filament remodelling induced by MAMP treatments is likely due to the negative regulation of CP by PA signalling.

**Figure 7:**
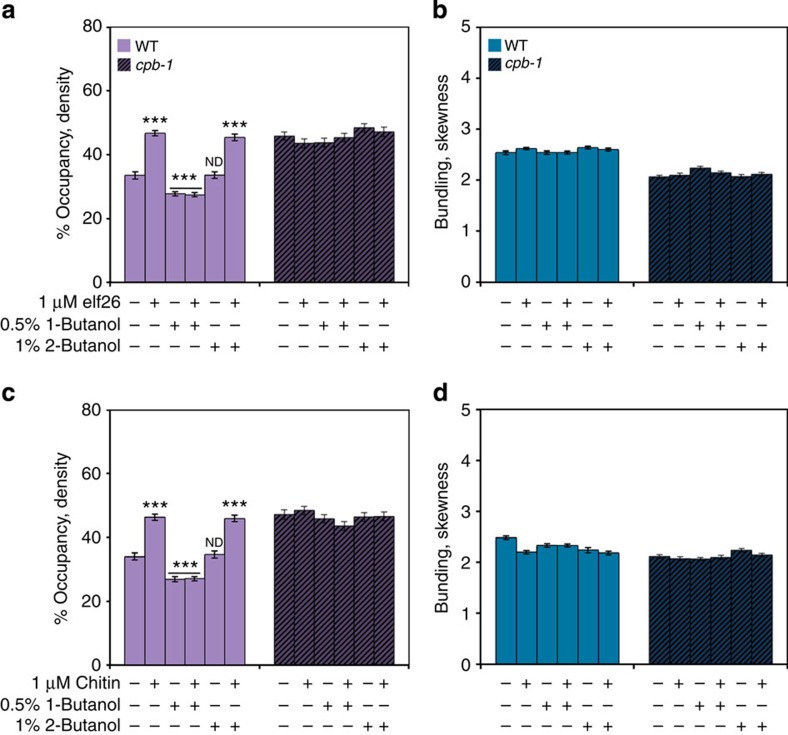
Effect of 1-butanol on cortical actin array remodelling following MAMP treatments. Percentage of occupancy or density was measured on epidermal cells of WT and *cpb-1* mutants treated with 0, 0.5% 1-butanol or 1% 2-butanol for 15 min, and followed by 5-min treatment with 1 μM elf26 (**a**) or 1 μM chitin (**c**). Treatments with 1-butanol significantly decreased the density of actin array in WT cells, as shown previously^29^. The actin abundance in WT cells treated with 1-butanol failed to increase following MAMP treatments. By contrast, actin filament abundance in the *cpb-1* mutants was not altered by either treatment. Treatments with 2-butanol had no measurable effects on actin filament levels in either WT or *cpb-1* seedlings. No significant differences in the extent of filament bundling were observed in WT and *cpb-1* mutant cells among treatments (**b**,**d**). Values given are mean±s.e.m. (*n*⩾200 cells from 10 hypocotyls for each treatment and genotype; ***P<0.001; ND, no significant difference compared with mock control of the same genotype; Student's *t*-test).

**Figure 8:**
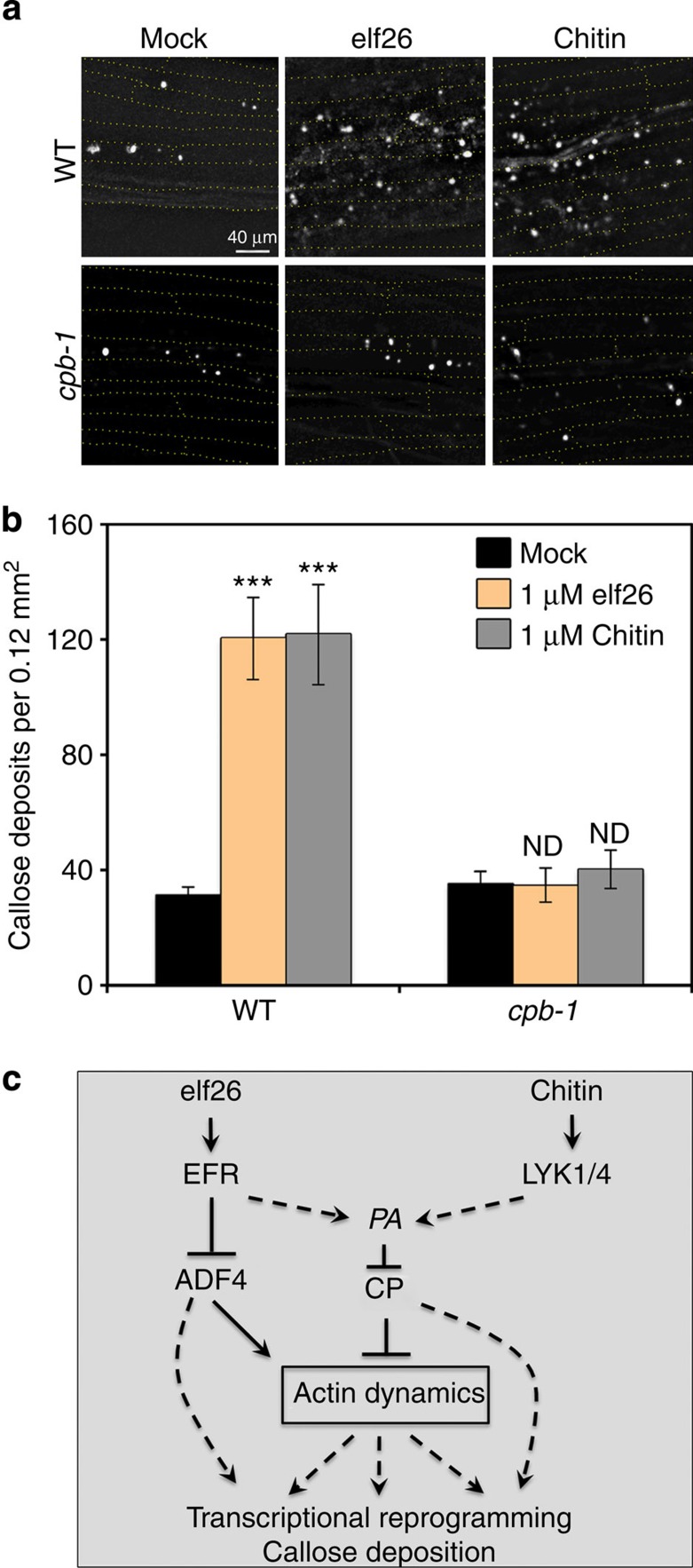
Increased callose deposition is not elicited by MAMP treatment of *cpb-1* cells. (**a**) Representative images show callose deposits in epidermal cells of WT and *cpb-1* mutant after mock, 1 μM elf26 and 1 μM chitin treatments. Scale bar, 40 μm. (**b**) Number of callose deposits indicated by aniline blue-stained spots was quantified in WT and *cpb-1* cells following mock, 1 μM elf26 and 1 μM chitin treatment. WT cells had significantly more callose deposits in response to treatment with MAMPs. Callose deposition failed to increase in *cpb-1* cells treated with MAMPs when compared with mock control. Values given are mean±s.e.m. (*n*=50 images per treatment, from at least 30 hypocotyls; ND, no significant difference from mock; ***P<0.001; Student's *t*-test). (**c**) Schematic model depicting the central role of CP in response to MAMP signalling. The perception of MAMPs by two different PRRs converges on PA signalling, which inhibits the activity of CP. However, ADF4 specifically functions downstream of the signalling pathway elicited by elf26 in dark-grown hypocotyls. The negative regulation of CP and ADF4 is required for rapid actin remodelling. Host defence responses, such as callose deposition and transcriptional reprogramming, may depend upon actin remodelling or could be due to actin-independent functions of CP and ADF4. (solid lines: direct interaction; dashed line: potential interaction; arrows: activation of signalling cascades; bars: inhibitory effect).

To test this hypothesis, we used the alcohol isomer, 1-butanol, to inhibit phospholipase D (PLD)-dependent PA production^29^,^31^. It has been shown previously that treatment of WT *Arabidopsis* epidermal cells with 1-butanol leads to a decrease in filament abundance in a dose-dependent manner^29^. As shown in Fig. 7a,c, WT cells treated with 1-butanol, at an intermediate concentration of 0.5% (v/v), had a significant decrease in filament abundance, which is consistent with previous results^29^. In addition, treatment of WT cells with 1-butanol completely suppressed the actin response after perception of either elf26 (Fig. 7a) or chitin (Fig. 7c), suggesting that PA production via the PLD pathway is essential for actin remodelling during MAMP signalling. By contrast, treatments with the inactive secondary alcohol, 2-butanol, at 1% (v/v) had no significant effect on actin architecture. Moreover, reduction of CP in the *cpb-1* mutant abrogated the effects of 1-butanol treatments when compared with WT cells (Fig. 7). Pretreament of hypocotyls with another PLD inhibitor, 5-fluoro-2-indolyl des-chlorohalopemide or FIPI^39^, gave similar results to 1-butanol (Supplementary Fig. 6). These data suggest that MAMP-stimulated actin remodelling results from the negative regulation of CP by PA/PLD signalling during plant innate immunity (Fig. 8c).

### Defence responses are impaired in the *cpb-1* mutant

MAMP perception by PRRs results in a variety of signalling responses with different temporal patterns. Rapid cellular events (seconds to minutes) include phosphorylation of MAPK and activation of MAPK signalling. Later events (minutes to hours) include extensive transcriptional reprogramming of defence-responsive gene networks. Long-term signalling and immune responses (hours to days) result in fortification of the cell wall by callose deposition^4^,^12^,^40^,^41^. Here we examined the requirement of CP for some of these readouts of innate immunity.

Callose deposition is a well-studied late cellular response to MAMP stimulation that requires an intact actin cytoskeleton^21^,^22^. To test whether CP is required for callose deposition, we analysed the number of callose deposits in epidermal cells from WT and *cpb-1* mutant hypocotyls. Following treatment with elf26 or chitin, WT cells showed a significant increase in callose deposition; however, the number of callose deposits in the *cpb-1* mutant failed to increase after treatment with either MAMP (Fig. 8a,b). These results demonstrate that CP plays an essential role in callose deposition elicited by elf26 and chitin treatment (Fig. 8c).

Upon MAMP perception, transcriptional reprogramming is mediated by synergistic or independent regulation of CDPK and MAPK signalling pathways^42^. In addition, different MAMPs activate both similar as well as different downstream defence genes^11^. It has also been shown that disruption of the actin cytoskeleton can affect transcriptional activation of defence-responsive genes^22^,^43^,^44^. Significantly, Henty-Ridilla *et al*.^22^ showed that loss of ADF4 only affects the transcriptional activation in the CDPK pathway following elicitation with elf26. Gene activation through the MAPK pathway, as well as chitin-induced transcriptional reprogramming, is still intact in the *adf4* mutant^22^, suggesting that different MAMP signalling networks require unique actin arrays with specific dynamic properties. To investigate whether reduction of CP inhibits transcriptional reprogramming in response to MAMP perception, we quantified the transcript levels for several genes that are responsive to MAMP-activated MAPK or CDPK signalling pathways^11^. These include MAPK-specific reporter gene, *FRK1*; MAPK-dominant pathway genes, *CYP81F2* and *WRKY33*; CDPK-specific response gene, *PHI1*; and the CDPK-synergistic pathway gene, *NHL10* (ref. 11). For most of these genes, no significant differences in transcript levels were detected between untreated *cpb-1* mutant and WT. However, the *cpb-1* mutant had significantly reduced transcript levels of *FRK1* and enhanced *PHI1* expression compared with WT (Supplementary Fig. 7). Following 1 h of elf26 or chitin treatment, the expression of all of these genes was significantly elevated in WT seedlings, which is consistent with previous results^11^,^22^. In the *cpb-1* mutant, however, the mRNA levels of these transcripts were not induced by chitin treatment; whereas all of the genes tested, except for *FRK1*, still responded to elf26 treatment (Supplementary Fig. 8). These data indicate that CP is upstream of transcriptional reprogramming in both chitin-triggered MAPK- and CDPK-dependent pathways, but is not responsible for the induction of gene expression in elf26-activated CDPK- or MAPK-dominant signalling pathways. Moreover, CP and ADF4 make distinct contributions to transcriptional reprogramming, suggesting that there are probably multiple defence processes and signalling pathways coordinated by several actin-binding proteins during innate immunity (Fig. 8c).

### CP enhances resistance to fungal and bacterial pathogens

**Figure 9:**
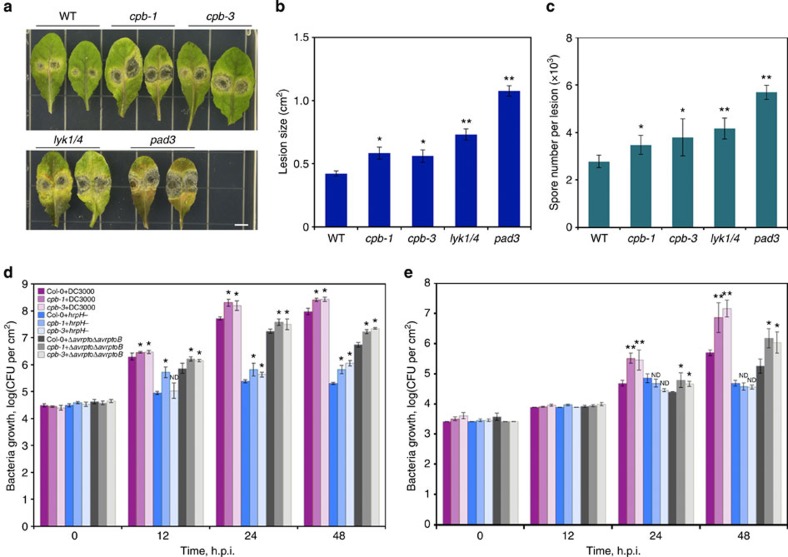
CP contributes to plant resistance against fungal and bacterial pathogens. (**a**–**c**) Plants of indicated genotypes were inoculated with *A. brassicicola*. Lesion size and spore number were measured 3 days post inoculation. Scale bar, 5 mm. (**d**,**e**) Bacterial growth with three *P. syringae* strains on WT, *cpb-1*, and *cpb-3* mutants at 0, 12, 24 and 48 h post infection (h.p.i.). Leaves were hand infiltrated with bacterial suspensions at 1 × 10^5^ colony-forming units (CFU) per ml (**d**) or dipped at 5 × 10^5^ CFU per ml (**e**). Values given are mean±s.e.m. (*P<0.01, **P<0.001; ND, no significant difference compared with WT control treated with same bacteria; Student's *t*-test).

To investigate the possible role of CP in plant resistance to microbial pathogens, we inoculated homozygous *cpb-1* and *cpb-3* mutants with the necrotrophic fungal pathogen *Alternaria brassicicola*. After infection, more severe disease symptoms were observed on leaves of the *cp* mutants compared with WT leaves inoculated with *Alternaria* (Fig. 9a). There was also a significant increase in lesion size and spore number after infection of *cp* mutants with *Alternaria* compared with WT (Fig. 9b,c). The increased susceptibility to *A. brassicicola* was also observed for *lyk1/4* and *pad3* mutants, which is consistent with previous results^8^,^9^. We further analysed whether *cp* mutants affect growth of different *Pseudomonas syringae* bacterial strains. Leaves from WT and the two *cpb* mutants were infected with pathogenic *P. syringae* pv. *tomato* DC3000 (DC3000), nonpathogenic type III secretion system-deficient (T3SS)-mutant *hrpH*, as well as the isogenic hypovirulent strain harbouring a deletion in *AvrPto* and *AvrPtoB (*Δ*avrpto*/Δ*avrptoB*). AvrPto and AvrPtoB are bacterial virulence factors that suppress early PTI signalling^45^. The growth of pathogenic and nonpathogenic bacteria on plants is likely to reveal phenotypes linked to PTI defects. As shown in Fig. 9d, when inoculated by hand infiltration of rosette leaves, plants with reduced CP levels showed enhanced susceptibility to all bacterial strains tested compared with growth on WT plants. Similarly, in experiments using dip inoculation, the *cp* mutants supported significantly greater growth of DC3000 and Δ*avrpto*/Δ*avrptoB* (Fig. 9e); however, the *hrpH* mutant did not show a difference in growth when grown on either genotype. The differences in bacterial growth between two types of inoculation suggest an involvement of plant stomatal defence. Bacterial pathogen entry into host tissues is the first step of infection. In *Arabidopsis*, it has been shown that stomatal closure is necessary for limiting bacterial invasion^46^. The restrictive role of stomatal closure on bacteria proliferation was more effective against bacteria inoculated onto the leaf surface than ones artificially infiltrated into the intercellular spaces^46^. Thus, the greater growth of *P. syringae* DC3000 strain on *cp* mutants infected by dip inoculation (Fig. 9e), compared with hand infiltration (Fig. 9d), hints that CP may play a role in stomatal defence. Taken together, these data suggest that CP plays a role in plant resistance to both fungal and bacterial pathogens.

## Discussion

In this study, we characterized the rapid remodelling of cortical actin arrays in response to MAMP signalling using epidermal cells from *Arabidopsis* dark-grown hypocotyls. Notably, in response to a diverse set of MAMPs, we observed an increase in actin filament abundance within minutes of perception. The results are similar to, but much faster than, the response of epidermal pavement cells from *Arabidopsis* cotyledons following MAMP treatments^18^. By combining high spatiotemporal imaging and novel metrics to quantify cytoskeletal dynamics with powerful genetic tools, the dark-grown hypocotyl has emerged as an ideal system to study actin dynamics in response to innate immune signalling^22^. Signals transmitted by several distinct PRR complexes trigger similar actin remodelling events, suggesting that the actin cytoskeleton response is a convergence point for basal defence machinery. Using genetic approaches, we demonstrated that CP integrates multiple MAMP signalling events that coordinate broad actin-dependent defence responses during innate immunity.

Perception of diverse MAMPs (for example, flg22, elf26 and chitin) by distinct PRR complexes results in an increase in actin filament abundance in both leaf epidermal cells and dark-grown hypocotyls^18^,^22^. Previously, we showed that perception of elf26 by the EFR PRR functioned through ADF4 resulting in actin remodelling in epidermal cells from dark-grown hypocotyls^22^. The *adf4* mutant responds normally to treatments with chitin, however, indicating that chitin signalling occurs independently of ADF4 (ref. 22). Here we provide genetic evidence that CP functions in multiple innate immune signalling pathways including the elf26-EFR and chitin-LYK1 pathways in hypocotyls.

CP is required for numerous actin-dependent processes in eukaryotic cells, several of which are also elicited during innate immunity. For example, receptor-mediated endocytosis, cell migration and lamellipodial protrusion are important for immune cell function in response to pathogenic stimuli^3^. Our data from plant cells show that actin arrays in knockdown mutants of CP are unresponsive to multiple MAMP stimuli. In addition, actin-dependent defence responses such as cell wall fortification and transcriptional reprogramming of defence-responsive genes are inhibited by reduction of CP. This could result from the inability of *cp* mutants to remodel actin networks or it might be due to the loss of an actin-independent function of CP. Nevertheless, we provide the first direct genetic evidence that CP manifests a link between innate immune signalling and remodelling of the actin cytoskeleton in plants.

The architecture and properties of actin arrays that support different cellular processes are highly unique. Investigating actin dynamics during innate immune signalling helps us gain deeper insight into the function of cytoskeletal regulators, new regulatory modes, as well as the temporal and mechanistic interplay between different classes of actin-binding proteins. Quantitative analyses of dynamic changes in the turnover of individual actin filaments following MAMP treatments have revealed the potential mechanisms that lead to increased filament abundance; these include downregulation of actin filament disassembly as well as an increase in free barbed ends for actin assembly^22^. Further, a previous study using pharmacological treatments suggests that actin polymerization also contributes to actin rearrangements during innate immunity^18^. Several actin-binding proteins have been revealed to regulate actin organization and dynamics in response to innate immune signalling^22^. For example, reduced actin filament turnover requires the inhibition of ADF4 or ADF1 severing activity^22^, whereas inhibition of CP leads to an increase in the number of barbed ends available for assembly.

In addition to the inhibition of several different actin-binding proteins by innate immune signalling, our work uncovered both parallel and convergent pathways for actin remodelling. Previously, we demonstrated that ADF4 plays a role in elf26 signalling, but is not required for the signalling pathway elicited by chitin in hypocotyl epidermal cells^22^. However, CP (this study) and ADF1 (ref. 22) are implicated in both pathways. These data further demonstrate that the repertoire of mechanisms that control actin filament dynamics *in vivo* is more complex than previously appreciated. However, this should not be totally surprising, given the plethora of proteins necessary to coordinate complicated processes such as endocytosis and vesicle trafficking.

In plant cells, MAMP perception by PRRs results in rapid cellular signalling events that occur on timescales of seconds to minutes, including cytosolic Ca^2+^ fluxes, phosphorylation of MAPK, and accumulation of ROS and signalling phospholipids^40^,^47^,^48^. These fast signals could impinge on the actin cytoskeleton by regulating the activity of actin-binding proteins, among which the regulation by phospholipids has been long recognized. PA is an abundant membrane phospholipid increasingly appreciated as an important signalling intermediate in plant cells^48^,^49^. PA can be generated either from the direct cleavage of structural phospholipids by PLD or through a series of reactions catalysed by phospholipase C and diacylglycerol kinase^31^. Cellular PA levels change rapidly in response to various biotic and abiotic stimuli, including pathogen attack^48^,^49^.

When elicited with MAMPs, PA accumulates within 2 min in tomato and rice suspension-cultured cells^50^,^51^. Pharmacological and genetic studies that inhibit PA production result in impaired production of ROS as well as a reduction in the antimicrobial molecules stimulated by elicitors^51^,^52^. In *Arabidopsis* leaf cells, exogenously applied PA is sufficient to induce ROS production and activation of defence-responsive genes^53^,^54^. Genetic evidence implicates PLDδ in nonhost resistance to powdery mildew fungus penetration^55^, whereas PLDβ is necessary for defence against bacterial pathogens^56^. These data demonstrate the important role of PA in innate immune signalling^57^. In this study, we further show that rapid actin remodelling in response to perception of MAMPs requires PLD-dependent PA signalling. Moreover, we provide genetic evidence that CP acts as a downstream target of PLD/PA signalling and a key transducer of fluxes in membrane signalling lipids into changes in actin cytoskeleton dynamics during plant innate immunity.

The actin cytoskeleton in plant cells responds not only to the perception of diverse MAMPs but it can also be a target for effector proteins that are injected by bacterial pathogens. Our previous study demonstrates that, during infection of *Arabidopsis* by *P. syringae* DC3000, increased actin filament bundling occurs at late time points, and is dependent on both T3SS and bacterial T3SS effectors^18^. However, it is unclear which effectors are involved in this actin rearrangement^18^. Recently, Kang *et al*.^58^ demonstrated that HopW1 from *P. syringae* pv. *maculicola* directly targets and disrupts actin filaments *in vitro* and *in vivo*^58^. Various actin-dependent processes, such as endocytosis and protein trafficking to the endoplasmic reticulum and/or vacuole, are impaired by ectopic expression of HopW1 in plant cells. In addition, the effect of HopW1 on actin cytoskeleton is necessary for promoting bacterial virulence^58^. These data further support the argument that the actin cytoskeleton is an essential component of host defence machinery involved in multilayered responses during dynamic plant–microbial interaction.

Our study and others show that blocking actin remodelling affects the transcriptional reprogramming of defence-responsive gene networks during plant innate immunity^22^,^43^,^44^,^59^. However, we have only a limited understanding of the underlying mechanisms. During the plant response to fungal infection, migration of the nucleus close to the penetration site requires an intact actin cytoskeleton in host cells^21^,^60^, and it associates with the activation of defence genes^60^. Moreover, actin and actin-binding proteins in the nucleus have been shown to regulate transcription and gene expression^61^,^62^. A nuclear-localized LIM domain protein from tobacco (NtWLIM2) directly binds to a histone gene promoter *in vitro* and *in vivo*, and activates histone gene expression^63^. In addition, actin-related proteins (ARP4–ARP9) are found in the nucleus of *Arabidopsis* cells^64^. Their homologues in yeast and animal cells are essential components of chromatin-remodelling complexes, suggesting a role in chromatin-mediated gene expression^62^. AtARP6 has been shown to activate flowering locus C expression through both histone acetylation and methylation^65^. It is also required for proper deposition of the H2A.Z histone variant into chromatin to modulate subsequent gene expression^66^. These data suggest that the plant actin cytoskeleton and specific actin-binding proteins may play a general role in gene expression and translation.

Hypothetically, actin remodelling triggered by MAMP signalling may be communicated to the genome in the following ways: changes in the ratio between filamentous and monomer actin in the cytoplasm could affect nuclear actin levels. MAMP signalling or subsequent actin rearrangement could lead to the release of actin-binding proteins and cytoskeleton-associated transcription factors, such that they are translocated into the nucleus and modulate gene expression. The latter has been suggested by the study of Porter *et al*.^44^ in which they show that nonphosphorylatable and phosphomimic mutants of ADF4 localize to the plant cell nucleus upon MAMP perception^44^. The nuclear translocation of ADF4 may correlate with attenuated gene activation through a CDPK pathway^22^. How CP affects transcriptional reprogramming during innate immunity, however, is rather complicated. Even though there is no direct evidence for nuclear CP, we cannot exclude the possibility that nuclear translocation of CP modulates the activity of transcription factors or chromatin-remodelling complexes. In addition, the impaired gene activation in *cp* mutant also likely results from the complex crosstalk between cellular signals, other cytoskeletal response regulators and various transcription factors that are associated with actin rearrangements. It is noteworthy that mutants for the *Arabidopsis* vesicle-trafficking protein dynamin-related protein 2B also had a differential response to flg22-elicited transcriptional programming^67^.

## Methods

### Plant material and growth conditions

The homozygous *lyk1* (096F09; also known as *cerk1–2*; refs 7, 8) and *lyk4* (WiscDsLox297300_01C; ref. 9) single mutants were crossed to WT *Arabidopsis thaliana* Col-0 expressing the GFP-fABD2 reporter^32^,^34^ and homozygotes were recovered from F2 populations. The homozygous *lyk1 lyk4* double mutant was transformed with a binary vector for the actin reporter *GFP-fABD2* by the floral-dip method^68^ and selected on kanamycin plates. T3 plants were used for analysis of actin organization. The *cpb-1* (SALK_014783), *cpb-3* (SALK_101017) and *efr* (SALK_044334) homozygous mutants expressing GFP-fABD2 were isolated and characterized previously^18^,^22^,^29^. Surface-sterilized seeds were plated onto 0.5 × Murashige and Skoog medium supplemented with 1% Suc and stratified at 4 °C for 3 days. After exposure to white light for 4 h, stratified seeds were grown in continuous darkness at 21 °C for 5 days.

### Plant treatments

Before use, elf26 (NeoBioSci, Cambridge, MA) or chitin (C9752, Sigma-Aldrich, St Louis, MO) was diluted in 1 × PBS at various concentrations. FIPI was purchased from Santa Cruz Biotechnology (Dallas, TX). Five-day-old etiolated seedlings were treated with butanol FIPI before MAMP treatments. For phospholipid treatments, 5-day-old dark-grown seedlings were soaked with PA or phosphatidlyserine micelles for 30 min before imaging^29^. As a negative control, seedlings were treated with an equivalent amount of solvent used to dilute the elicitors.

### Quantitative analyses of cortical actin array architecture

The cortical actin array in epidermal cells was imaged using VAEM on an inverted microscope with an Olympus TIRF illuminator^32^. Epidermal cells from the basal third of 5-day-old dark-grown hypocotyls expressing GFP-fABD2 were documented with a series of overlapping VAEM micrographs. A fixed exposure time and gain settings were selected for all the genotypes or treatments and their respective controls. Skewness analysis was used to monitor the extent of actin filament bundling, and the filament density was calculate as the percentage of occupancy of GFP-fABD2 signal in each micrograph^22^,^29^,^35^,^36^,^37^. Micrographs were analysed with ImageJ (http://rsb.info.nih.gov/ij/) using Higaki's macro available at http://hasezawa.ib.k.i-tokyo.ac.jp/zp/Kbi/HigStomata^29,35,37^. All data analyses were performed as double-blind experiments.

### Time-lapse imaging of actin filament dynamics

The cortical actin array in WT and *cpb-1* mutant epidermal cells expressing GFP-fABD2 was recorded by time-lapse VAEM^29^,^32^,^37^. Following treatment with 1 μM MAMPs, epidermal cells from the basal third of 5-day-old dark-grown hypocotyls were examined. For elongation rate analysis, filament length was plotted over time. Rates were determined as the slope of lines that fit the best linear function. Maximum filament lifetime was the total duration when each filament was present. Maximum filament length was the longest length of a tracked filament during the course of growth and turnover. Severing frequency was estimated by measuring maximum length of each filament and recording subsequent breaks over time. Regrowth or annealing of severed ends was determined by dividing the number of events observed with the total number of ends for each filament. Filament origin was defined as a percentage of the first 10–15 filaments observed per cell that originated from indicated locations^22^,^29^,^32^,^37^. For correlation coefficient analyses, time-lapse VAEM series were cropped and analysed using the built-in MATLAB function corr2 defined by Vidali and colleagues^30^,^38^.

### Callose staining

For analysis of MAMP-stimulated callose deposits, 5-day-old dark-grown seedlings were soaked in 1 μM MAMPs for 16 h before aniline blue staining^22^,^69^. Callose spots in cells from the base of the hypocotyl were observed using Nikon E600 epifluorescence microscope equipped with a × 20 0.5-numerical aperture PlanFluor objective under ultraviolet light. Images were captured with a CCD camera (ORCA-ER C4742-95; Hamamatsu Photonics) and Metamorph software (Version 4.6r9, Molecular Devices, Sunnyvale, CA). The mean number of callose spots present in a 0.12-mm^2^ region of interest in each cell was quantified using ImageJ.

### RNA extraction and Real-Time quantitative PCR (RT-qPCR)

Five-day-old etiolated seedlings were collected and flash frozen in liquid nitrogen. Total RNA was isolated using TRIzol reagent (Invitrogen) according to the manufacturer's instructions. The isolated RNA was further treated with RQ1 DNase (Promega) to remove potential DNA contamination. cDNA was synthesized using M-MLV reverse transcriptase according to manufacturer's instructions (Invitrogen). Two-step RT-qPCR was performed using 2 × SYBR Green master mix (Qiagen). To analyse the expression of *LYK1* and *LYK4*, gene-specific primers as given in Supplementary Table 2 were used^8^,^9^. To quantify the transcriptional reprogramming that occurs during the defence response, 5-day-old etiolated seedlings of WT and *cpb-1* mutant were soaked in the presence or absence of 1 μM MAMPs for 1 h before RNA extraction. Specific primers for the MAPK and CDPK pathway^11^ genes used in this study are given in Supplementary Table 2 (ref. 22). *GAPD* was used as an internal control to normalize gene expression across different samples. The fold induction in the target gene, normalized to *GAPD* and relative to the gene expression in the control sample, was calculated using comparative ΔΔCt (ref. 36). Three biological and technical replicates were performed per treatment.

### Disease assays

For plant infection with *A. brassicicola*, each rosette leaf was inoculated with one or two droplets of 10 μl spore suspension at a concentration of 5 × 10^4^ spore per ml. Thirty to forty leaves were collected after 3 days for each genotype. The spores were washed from leaves and the number of spores were determined with a haemacytometer^9^. Experiments were repeated three times. To measure bacterial growth on *Arabidopsis*, briefly, 24-day-old leaves were hand infiltrated with bacterial suspensions at 1 × 10^5^ colony-forming units per ml using a needless syringe, or dip inoculated at 5 × 10^5^ colony-forming units per ml. Two leaf discs with a diameter of 0.5 cm^2^ were collected at various time points after infection and ground in 10 mM MgCl_2_. Following bacteria recovery, serial dilution of leaf extracts was performed. A 2-μl drop from each dilution was plated for counting bacterial colonies^18^,^70^. Each data point represents average bacterial numbers from three replicates.

## Additional information

**How to cite this article:** Li, J. *et al*. Capping protein integrates multiple MAMP signalling pathways to modulate actin dynamics during plant innate immunity. *Nat. Commun.* 6:7206 doi: 10.1038/ncomms8206 (2015).

## Supplementary Material

Supplementary Information Supplementary Figures 1-8, Supplementary Tables 1-2 and Supplementary References
