# Supplementary material for: Capping protein integrates multiple MAMP signalling pathways to modulate actin dynamics during plant innate immunity
Source: Nat Commun. 2015 May 28;6:7206. doi: 10.1038/ncomms8206 (PMC4458898; doi:10.1038/ncomms8206)
Supplement: Supplementary Information — Supplementary Figures 1-8, Supplementary Tables 1-2 and Supplementary References [file ncomms8206-s1.pdf]

Supplementary Information

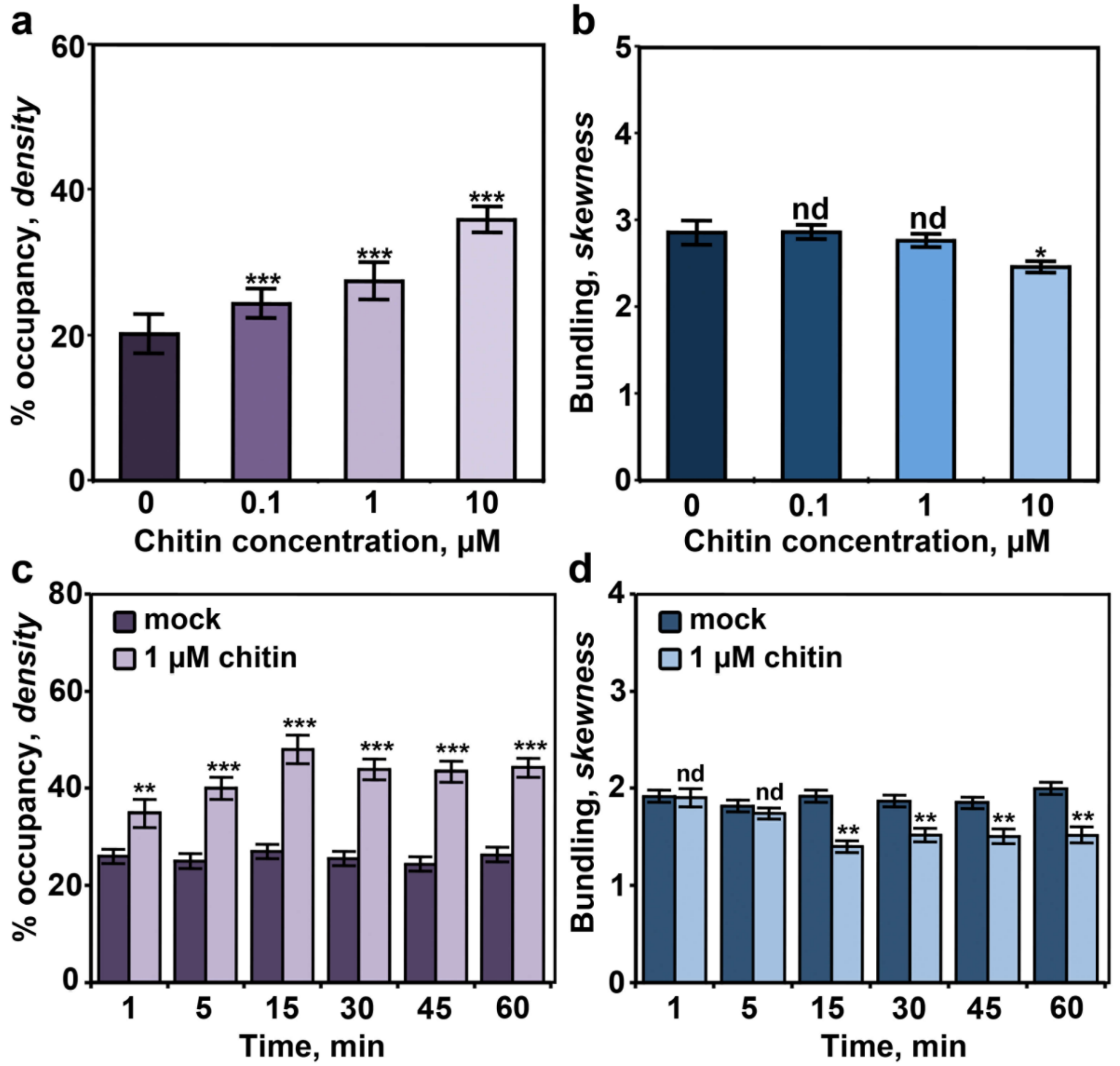

Supplementary Figure 1. The increase in actin filament abundance in response to chitin treatment is dose and time dependent. (**a**,**b**) Actin architecture analysis was performed on WT epidermal cells treated for 5 min with 0, 0.1, 1 and 10 µM chitin. The density of actin filament arrays was significantly increased by chitin in a dose-dependent fashion (**a**). A slight decrease in the extent of filament bundling occurred when cells were treated with 10 µM chitin (**b**). (**c**,**d**) The increase in actin filament density occurred as early as 1 min after treatment with 1 µM chitin and reached a maximum level after 15 min (**c**). Actin arrays were significantly less bundled after MAMP treatments > 15 min (**d**).
Values given are means ± s.e.m ($n \geq 200$ cells from 10 hypocotyls for each treatment per timepoint; nd, no significant difference; **P <0.05; **P < 0.01; ***P < 0.001 compared to respective mock control; Student's *t* test).

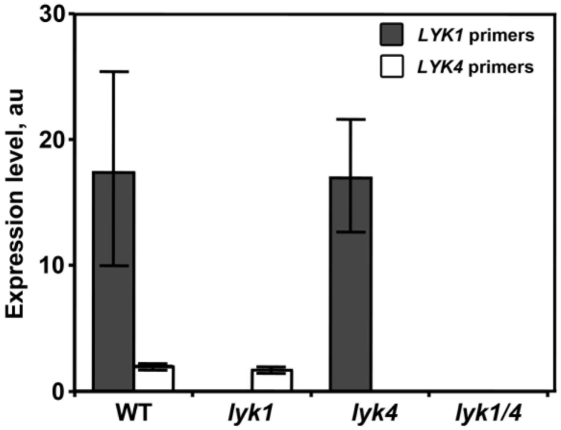

Supplementary Figure 2. *LYK1* and *LYK4* are expressed in dark-grown *Arabidopsis* seedlings. The transcript levels for *LYK1* and *LYK4* were quantified by RT-qPCR analysis on dark-grown seedlings of WT, *lyk1* and *lyk4* single mutants, as well as a *lyk1 lyk4* double mutant. The expression of *LYK1* was more abundant than *LYK4* in etiolated seedlings and was completely absent in *lyk1* mutant, whereas *LYK4* transcripts remained similar to WT seedlings. No *LYK4* transcripts were detected in *lyk4* mutant, whereas *LYK1* was expressed normally. The transcripts for both genes were absent in the *lyk1 lyk4* double mutant. Mean values from triplicate biological samples and technical replications are plotted ± s.e.m., normalized to GAPD expression.

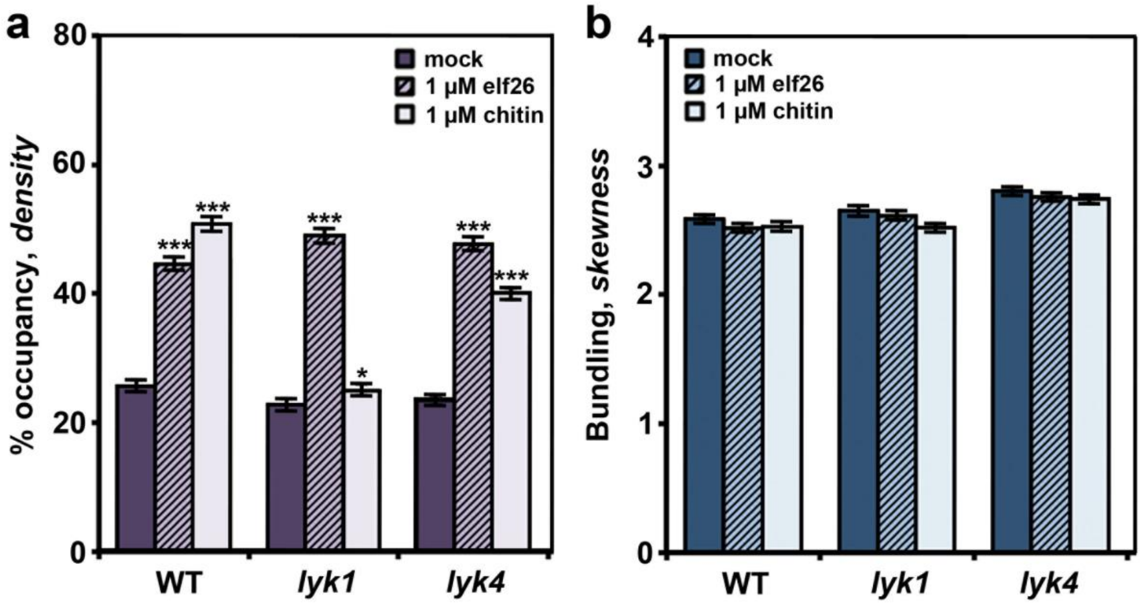

Supplementary Figure 3. LYK1 plays a major role in translating the perception of chitin into cytoskeleton remodeling. Actin architecture analysis was performed on hypocotyl epidermal cells of *lyk1* and *lyk4* single mutant seedlings following 5 min treatment with mock, 1 µM elf26 and 1 µM chitin, the wild-type siblings of *lyk1* mutant were used as positive controls. The density of actin filament arrays in WT cells was significantly increased in response to both MAMPs. When compared with mock control, only a minor increase in filament density was induced in the *lyk1* single mutant treated with chitin. The density of actin filament arrays was significantly increased following chitin treatment in the *lyk4* single mutant, but not as strongly as in chitin-treated WT cells. However, elf26 treatment stimulated an increase in the density of actin filament arrays that was comparable to WT in both *lyk* mutants (**a**). No significant change in filament bundling was observed in any genotype or treatment tested (**b**).
Values given are means ± s.e.m. ($n \geq 200$ cells from 10 hypocotyls for each treatment and genotype; *$P < 0.05$; ***$P < 0.001$ compared to mock control of the same genotype; Student's *t* test).

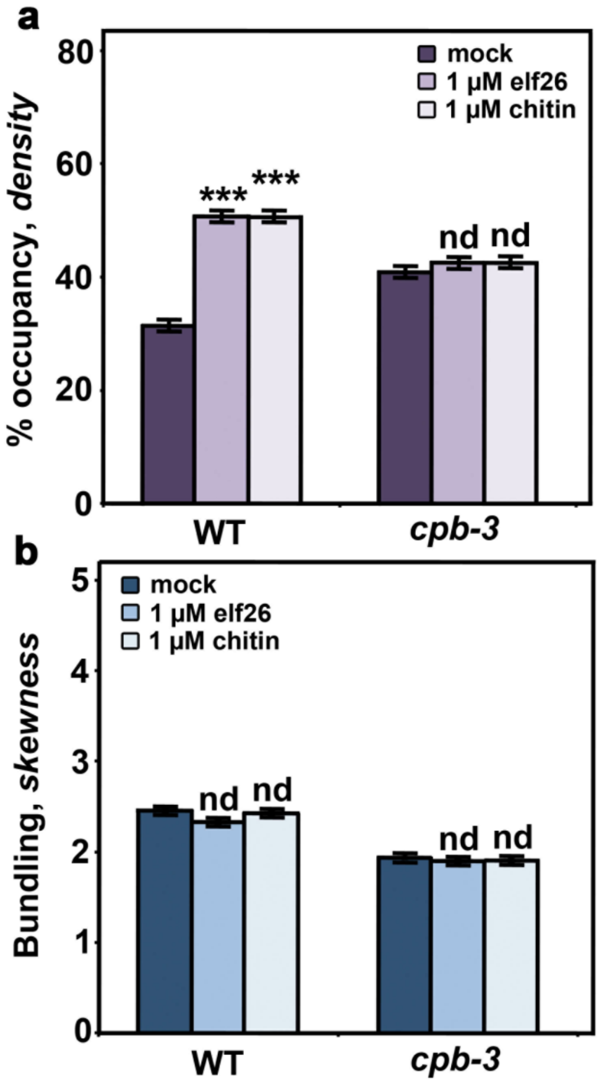

Supplementary Figure 4. Actin architecture in *cpb-3* mutant cells fails to respond to both chitin and elf26 treatments.

Actin architecture analysis was performed on epidermal cells of WT and *cpb-3* mutant treated with mock and MAMPs for 5 min. The cortical actin array in mock-treated *cpb-3* mutant cells was more dense compared to WT cells, as shown previously[1,2]. Following MAMP treatment, the actin filament abundance in WT cells was significantly enhanced, whereas no significant changes in actin filament abundance were detected in *cpb-3* mutant cells treated with elf26 or chitin compared to mock control (**a**). The extent of filament bundling was not altered in WT and *cpb-3* mutant cells after MAMP treatments compared with their respective mock controls (**b**).

Values given are means ± s.e.m. ($n \geq 200$ cells from 10 hypocotyls for each treatment and genotype; ***$P < 0.001$; nd, no significant difference compared to mock control of the same genotype. Student's *t* test).

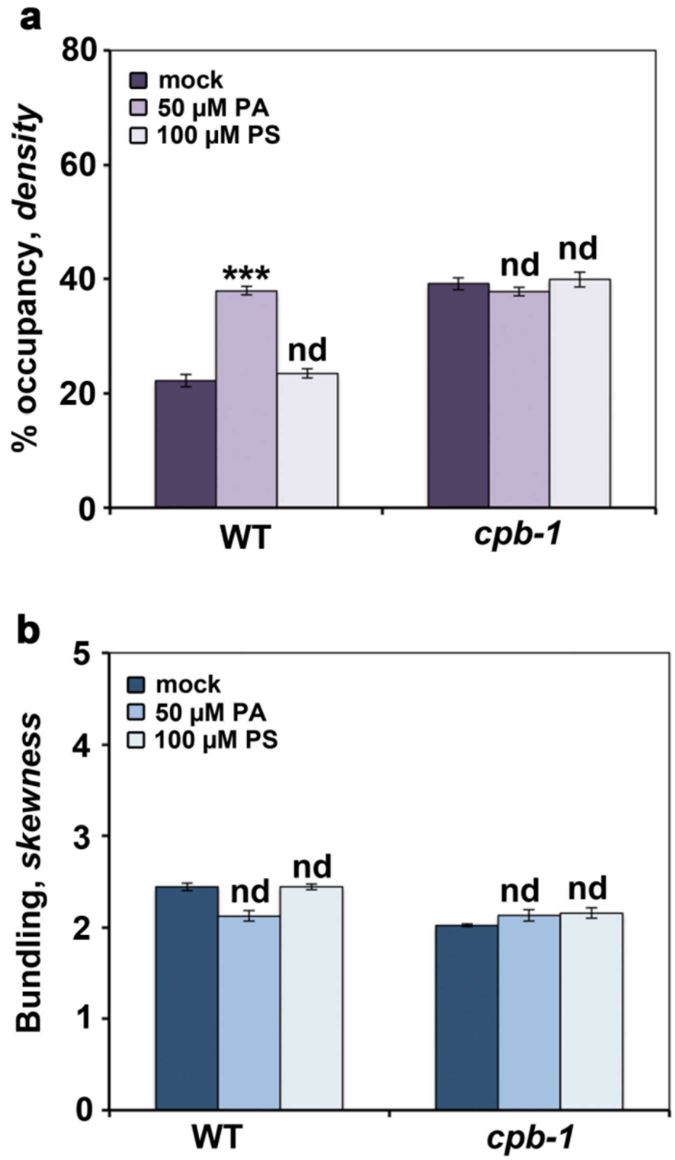

Supplementary Figure 5. Reduction of CP blocks the PA-induced increase in filament abundance.

Actin architecture analysis was performed on epidermal cells of WT and *cpb-1* mutant treated with 0, 50 µM PA or 100 µM PS for 30 min, as described previously[1]. WT cells treated with PA had a significant increase in filament abundance, whereas PA treatment of the *cpb-1* mutant did not increase filament density. Treatments with PS had no measurable effects on actin filament levels in either WT or *cpb-1* seedlings (**a**). No significant differences in the extent of filament bundling were observed in WT and *cpb-1* mutant cells among treatments (**b**).

Values given are means ± s.e.m. ($n \geq 200$ cells from 10 hypocotyls for each treatment and genotype; ***$P < 0.001$; nd, no significant difference compared to mock control of the same genotype. Student's *t* test).

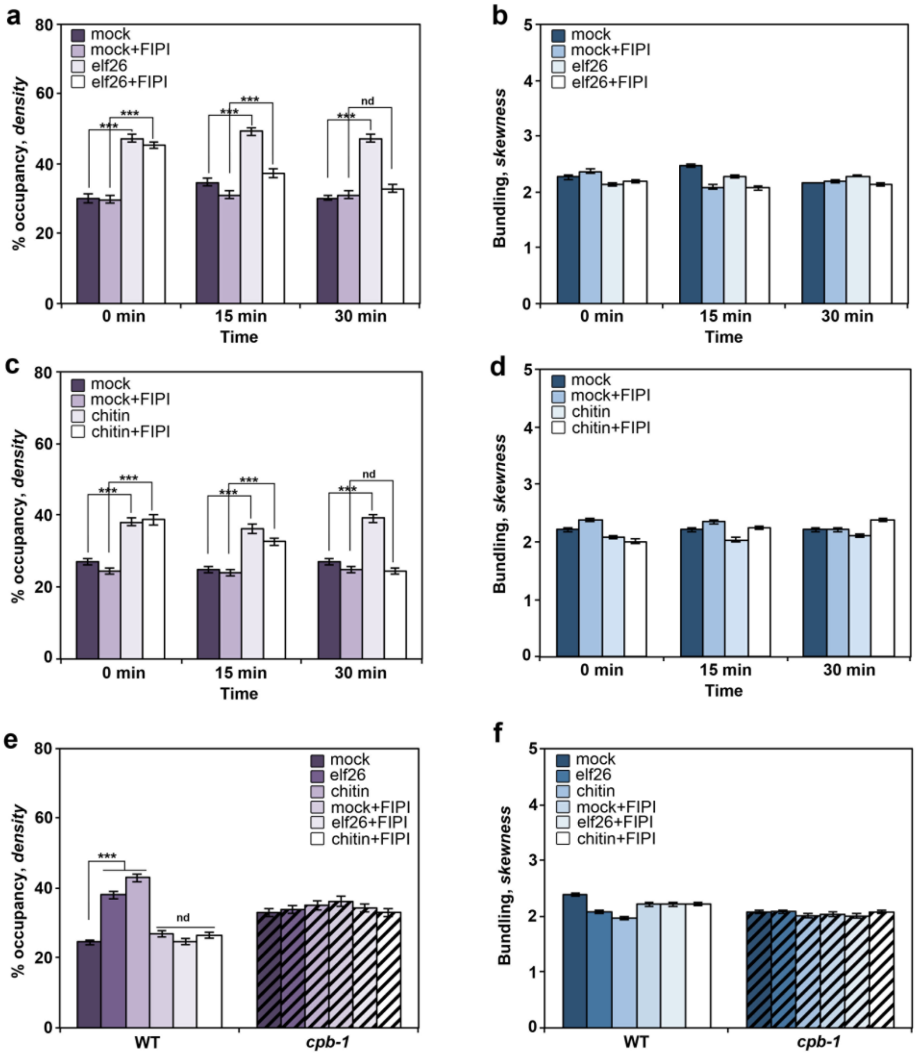

Supplementary Figure 6. FIPI treatment blocks MAMP-triggered actin remodeling.

Percentage of occupancy or *density* was measured on epidermal cells of WT (**a,c,e**) or *cpb-1* mutant (**e**) treated with 750 nM FIPI for different time periods (30 min in [**e**]), and followed by 5-min treatment with 1 µM elf26 or 1 µM chitin. Pretreatments with the PLD inhibitor 5-fluoro-2-indolyl des-chlorohalopemide (FIPI) in the absence of MAMP stimulation had no significant effect on the density of actin arrays in WT cells when compared with mock control. However, FIPI inhibited the increase in actin filament abundance following MAMP treatments, in a time-dependent manner (**a,c**). By contrast, actin filament abundance in the *cpb-1* mutant was not altered by either treatment (**e**). No significant differences in the extent of filament bundling were observed in WT and *cpb-1* mutant cells among the various treatments (**b,d,f**).

Values given are means ± s.e.m. ($n \geq 200$ cells from 20 hypocotyls for each treatment and genotype; ***$P < 0.001$; nd, no significant difference. Student's *t* test).

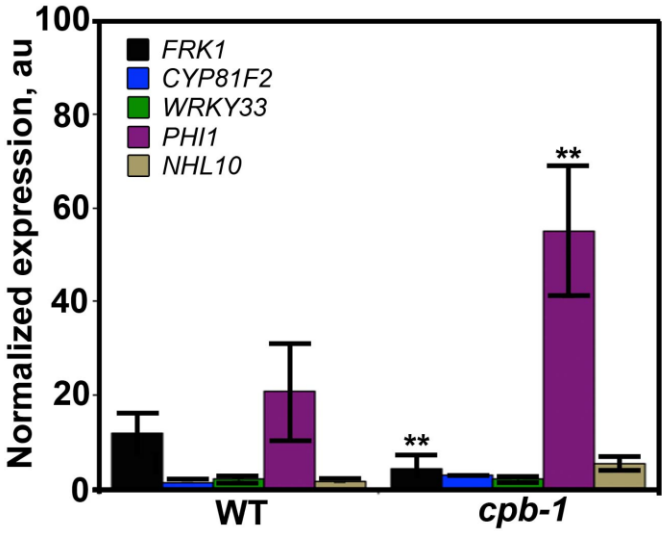

Supplementary Figure 7. Basal expression levels of defense responsive genes in WT and *cpb-1* mutant. The basal expression level of defense responsive genes was monitored by real-time quantitative PCR (RT-qPCR) of untreated, dark-grown WT and *cpb-1* mutant seedlings. The transcript levels of MAPK-specific reporter gene, *FRK1*, as well as MAPK dominant-pathway genes *CYP81F2* and *WRKY33*; A CDPK-specific response gene, *PHI1* and the CDPK-synergistic pathway gene, *NHL10* were tested. Mean values from triplicate biological samples and technical replications were normalized to *GAPD* expression in the same genotype and plotted ± s.e.m., (**P < 0.01; Student's *t* test).

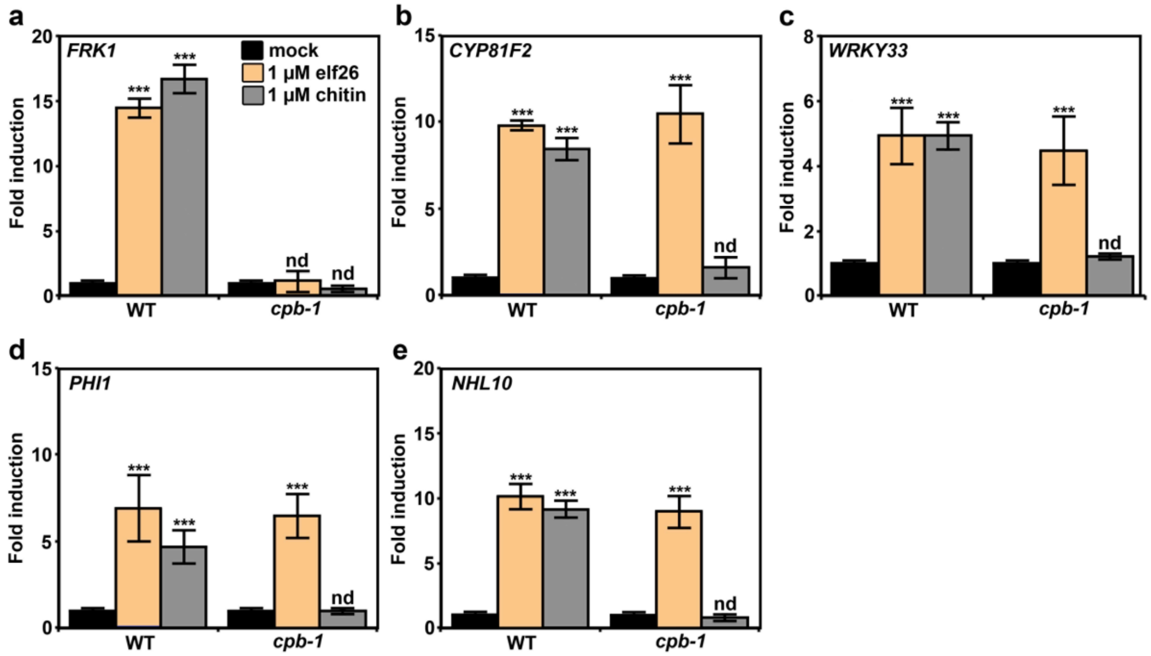

Supplementary Figure 8. Transcriptional activation of CDPK and MAPK pathways is altered in *cpb-1* mutant following MAMP treatments. Quantitative analysis of marker genes for initial defense signaling. In WT plants, the transcript levels of defense responsive genes: *FRK1* (**a**), *CYP81F2* (**b**), *WRKY33* (**c**), *PHI1* (**d**) and *NHL10* (**e**) were activated by elf26 or chitin treatments. Except for *FRK1*, the expression of these defense-induced genes was not altered in *cpb-1* mutant treated with elf26. The induction of all the MAMP-responsive genes tested was impaired in *cpb-1* mutant following chitin treatment. Mean values from triplicate biological samples and technical replications are plotted ± s.e.m., normalized to *GAPD* expression and presented as fold induction from mock (**P < 0.01; ***P < 0.001; nd, no significant difference compared to mock control of the same genotype; Student's *t* test).

Supplementary Table 1. Actin dynamic parameters from MAMP-treated WT and *cpb-1* mutant epidermal cells

		WT			*cpb-1*		
		**Mock**	**1 μM elf26**	**1 μM chitin**	**Mock**	**1 μM elf26**	**1 μM chitin**
Elongation rate; μm s^{-1}		1.7 ± 0.1	1.8 ± 0.1[nd]	1.7 ± 0.1[nd]	1.8 ± 0.1	1.8 ± 0.1[nd]	1.9 ± 0.1[nd]
Severing frequency; breaks $μm^{-1}$ s^{-1}		0.015 ± 0.007	0.010 ± 0.001[b]	0.010 ± 0.001[b]	0.012 ± 0.005[a]	0.011 ± 0.001[nd]	0.011 ± 0.001[nd]
Max. filament length; μm		11.9 ± 0.5	17.8 ± 0.6[b]	19.3 ± 0.5[b]	15.1 ± 0.5[a]	16.7 ± 0.5[nd]	16.0 ± 0.5[nd]
Max filament lifetime; s		19.7 ± 0.8	25.1 ± 0.9[b]	24.2 ± 0.9[b]	26.1 ± 1.2[b]	26.0 ± 1.1[nd]	28.9 ± 1.1[nd]
Re-growth of severed ends; %		2.8 ± 1.2	2.7 ± 1.1[nd]	4.3 ± 1.5[nd]	3.7 ± 1.4	4.4 ± 1.6[nd]	2.7 ± 1.1[nd]
Annealing of severed ends; %		2.4 ± 1.2	8.9 ± 2.4[a]	10.1 ± 2.8[a]	9.5 ± 2.3[a]	10.4 ± 2.6[nd]	11.1 ± 2.4[nd]
Filament origin; % per cell	*de novo*	34.3 ± 2.6	26.7 ± 2.1	24.7 ± 2.5	26.3 ± 2.3	27.0 ± 2.2	30.6 ± 2.8
	ends	21.7 ± 2.5	20.0 ± 2.6[c]	36.7 ± 4.2[c]	41.3 ± 3.5[c]	34.7 ± 3.6[d]	34.7 ± 3.6[d]
	side	44.0 ± 3.1	53.3 ± 2.7	38.7 ± 3.7	32.3 ± 2.5	38.3 ± 3.6	34.3 ± 3.1

Values given are mean ± s.e.m., with n > 50 filaments from n > 10 epidermal cells and at least 10 hypocotyls per treatment.
For filament origin: n > 30 cells from at least 10 hypocotyls per treatment.
[nd]Not significantly different from mock control value of the same genotype by Student's t test; P > 0.05.
[a]Significantly different from WT mock control value by Student's t test; P < 0.01.
[b]Significantly different from WT mock control value by Student's t test; P < 0.001.
[c]Significantly different from WT mock control value by ANOVA; P < 0.05.
[d]Significantly different from *cpb-1* mock control value by ANOVA; P < 0.05.

Supplementary Table 2. Gene-specific primers used for RT-qPCR.

Gene	Forward Primer	Reverse primer
LYK1	5'-AGAATATATCCACGAGCACACGGTTCCAG-3'	5'-GACGAAAAGAGAGTGGATAAAGCAACCAC-3'
LYK4	5’-CCACAATCGGTTTCTCCTCCTCCATTGTC-3’	5’-GTACGACGATTCTTCCCAGTTCTGCGTAG-3’
FRK1	5'-GGGTCAGATTTCAACAGTTGTC-3'	5'-AATAGCAGGTTGGCCTGTAATC-3'
WRKY33	5'-GTGATATTGACATTCTTGACGA-3'	5'-GATGGTTGTGCACTTGTAGTA-3'
PHI1	5'-TTGGTTTAGACGGGATGGTG-3'	5'-ACTCCAGTACAAGCCGATCC-3'
NHL10	5'-TTCCTGTCCGTAACCCAAAC-3'	5'-CCCTCGTAGTAGGCATGAGC-3'
CYP81F2	5'-AAATGGAGAGAGCAACACAATG-3'	5'-ATCGCCCATTCCAATGTTAC-3'
GAPD	5'-CACTTGAAGGGTGGTGCCAAG-3'	5'-CCTGTTGTCGCCAACGAAGTC-3'

Supplementary references

1 Li, J. *et al.* Capping protein modulates the dynamic behavior of actin filaments in response to phosphatidic acid in *Arabidopsis*. *Plant Cell* **24**, 3742-3754 (2012).

2 Li, J. *et al.* The availability of filament ends modulates actin stochastic dynamics in live plant cells. *Mol. Biol. Cell* **25**, 1263-1275 (2014).
